# An Implantable Cranial Window Using a Collagen Membrane for Chronic Voltage-Sensitive Dye Imaging

**DOI:** 10.3390/mi10110789

**Published:** 2019-11-18

**Authors:** Nobuo Kunori, Ichiro Takashima

**Affiliations:** Human Informatics Research Institute, National Institute of Advanced Industrial Science and Technology (AIST), Tsukuba 305-8568, Japan; n-kunori@aist.go.jp

**Keywords:** optical imaging, voltage-sensitive dye, atelocollagen membrane, artificial dura matter, 3D printer, implantable device

## Abstract

Incorporating optical methods into implantable neural sensing devices is a challenging approach for brain–machine interfacing. Specifically, voltage-sensitive dye (VSD) imaging is a powerful tool enabling visualization of the network activity of thousands of neurons at high spatiotemporal resolution. However, VSD imaging usually requires removal of the dura mater for dye staining, and thereafter the exposed cortex needs to be protected using an optically transparent artificial dura. This is a major disadvantage that limits repeated VSD imaging over the long term. To address this issue, we propose to use an atelocollagen membrane as the dura substitute. We fabricated a small cranial chamber device, which is a tubular structure equipped with a collagen membrane at one end of the tube. We implanted the device into rats and monitored neural activity in the frontal cortex 1 week following surgery. The results indicate that the collagen membrane was chemically transparent, allowing VSD staining across the membrane material. The membrane was also optically transparent enough to pass light; forelimb-evoked neural activity was successfully visualized through the artificial dura. Because of its ideal chemical and optical manipulation capability, this collagen membrane may be widely applicable in various implantable neural sensors.

## 1. Introduction

A variety of implantable neural probes have been developed as core technology for brain–machine interface (BMI) systems [1,2]. Among them, the Utah array [3] is a popular micromachined electrode that has been successfully used for recording cortical neural activity in specific brain areas to collect BMI signals [4,5]. The Utah array-based BMI system has now been applied to human patients as cortical prostheses [6,7]. As is the case with array electrodes, the direct electrical measurement of neuronal activity is a promising technique for a BMI; however, another approach using an optical method has potential when incorporated with future BMI systems. Because of its indirect measurement principle, an optical neural probe is somewhat less invasive, and is expected to collect neural activity from a wide array of cortical areas at high spatiotemporal resolution.

Two possible candidates for optical probes are calcium (Ca) and voltage-sensitive dye (VSD) imaging. Some elaborate studies have reported fiber optic periscope [8] or epi-fluorescence microscopic [9] devices that allow stable Ca imaging of cortical neurons in freely-behaving animals. Ca imaging provides a less direct measure of spiking activity but can offer valuable information when combined with genetic methods to highlight the activity of targeted neuron populations. By contrast, VSD imaging directly monitors membrane potential dynamics [10], and a close relationship has been demonstrated between dye signals and spiking activity [11,12]. However, at least two problems have to be solved when applying the VSD imaging method as an implantable neural sensor. First, a dural substitute, which should be biocompatible, flexible, and nearly transparent, is necessary for long-term optical imaging. This is because a relatively large cortical surface must be exposed for VSD imaging and the dura mater must be removed. Second, a simple and convenient staining procedure must be established. The exposed cortex needs to be repeatedly stained with the dye and washed with artificial cerebrospinal fluid (ACSF).

Previous studies in non-human primates have employed the artificial dura method to solve these problems. They replaced the native dura with a transparent artificial dura (polyurethane sheet or silicone rubber) and attained long-term intrinsic (without dye) optical [13,14,15] and VSD imaging [16,17]. However, the artificial duras used were impermeable to dye; therefore, a thin tube inserted under the artificial dura was prepared as a staining inlet [16,17]. The extra tubing was troublesome when using an implantable neural sensor. In the present study, we used an atelocollagen membrane as artificial dura material, which is often used as a cell-culture substrate [18,19]. We fabricated the cranial window using a collagen membrane, implanted it in rats, and performed VSD imaging within 1 week. The results showed the following: (1) the collagen sheet worked as an artificial dura to protect the exposed cortex; (2) the dye passed through the collagen membrane and successfully stained the cortex; and (3) the collagen sheet was optically transparent enough to allow VSD imaging.

## 2. Materials and Methods

### 2.1. Implantable Cranial Window Device with Collagen Membrane

**Figure 1:**
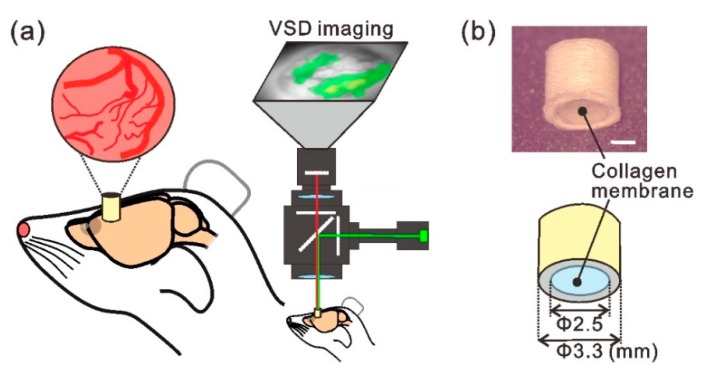
Experimental concept of the implantable cranial window for chronic voltage-sensitive dye (VSD) imaging. (**a**) Schematic view of the experimental setup. A cylinder-like device was attached to the skull and conventional VSD imaging [21] was performed by peering through it. (**b**) Upper image: photograph of the tube-shaped implantable-chamber device, wherein the bottom end of the tube was sealed with the atelocollagen membrane. The device was printed using a high-resolution three-dimensional printer. Lower image: design drawing of the device. Scale bar in (**b**), 1 mm.

The implantable chamber device was a tube-shaped acrylic structure with a length of 3.0 mm and inner and outer diameters of 2.5 and 3.3 mm, respectively (Figure 1b). We used FreeCAD 0.16 software to design the tube structure and fabricated it using a high-resolution three-dimensional (3D) printer (AGILISTA-3000; Keyence, Osaka, Japan). We used a semipermeable atelocollagen membrane sheet (CLF-01; KOKEN, Tokyo, Japan) as the dura substitute. Atelocollagen is a low-immunogenic derivative of type I collagen obtained by removing the N- and C-terminal telopeptide components and is regarded as a highly biocompatible biomaterial. The membrane sheet can be cut into any shape and size, and has a thickness of 35 μm. One end of the tube structure was sealed by the collagen membrane using biocompatible adhesive (Aron alpha A; Sankyo, Tokyo, Japan). The sealed side of the tube device was gently pressed against the cortical surface [20].

### 2.2. Animals and Surgical Procedures

This experiment was approved by the Committee on Animal Care Use and by the Ethical Committee of the National Institute of Advanced Industrial Science and Technology. Male Wistar rats (325 ± 25 g; SLC Inc., Tokyo, Japan) were used in all experiments (n = 7). The animals were group housed in a standard cage under a 12 h light/dark cycle with food and water provided ad libitum. The rats were anesthetized with isoflurane (3.0% induction, 1.50%–1.25% maintenance) and fixed in a stereotaxic frame for the experiments. A craniotomy was performed to create a circular cranial window (3.5 mm in diameter) over the secondary motor cortex (M2; anteroposterior, 3.0 mm; mediolateral, 1.0 mm from the bregma [22]). After removing the dura in the cranial window, the tube-shaped implantable device was carefully placed on the exposed cortex using a micro manipulator (SM-15, Narishige, Tokyo, Japan) and fixed to the skull surface using silicon adhesive (for packing) and dental acrylic cement (for fixing). After the surgery, the tube was filled with ACSF (124 mM NaCl, 2.5 mM KCl, 2 mM CaCl_2_, 2 mM MgSO_4_, 1.25 mM NaH_2_PO_4_, 26 mM NaHCO_3_, and 10 mM glucose) and sealed with silicon adhesive (Kwik-Sil; WPI, Sarasota, FL, USA).

### 2.3. Voltage-Sensitive Dye (VSD) Imaging

**Figure 2:**
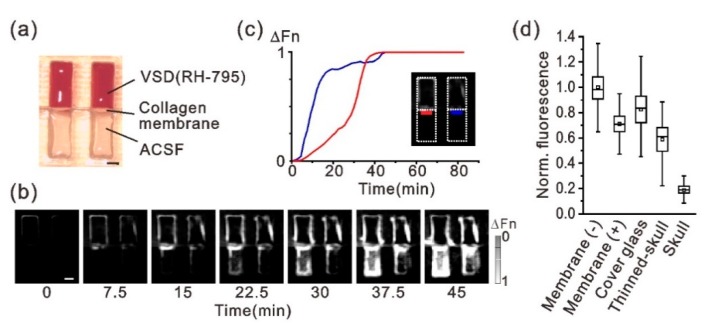
Chemical and optical transparency of the collagen membrane. (**a**) Photograph of two test tubes. The upper and lower parts of the tube well were separated by the collagen membrane; one part was filled with RH-795 VSD and the other with artificial cerebrospinal fluid (ACSF). Thereafter, time-lapse VSD imaging was conducted to show the time course of the diffusion of the VSD. (**b**) Time-lapse VSD images showing dye diffusion across the collagen membrane toward the ACSF. Each gray-scale frame indicates the change in dye fluorescence from t = 0. The gray-scale bar represents the peak-normalized ΔF/F value (ΔFn). (**c**) Time changes in the fluorescence amplitude measured near the collagen membrane on the side with ACSF solution. The traces are from pixels selected in the red and blue rectangular areas in the inset image. (**d**) Optical transparency of the collagen membrane. Fluorescence intensity from the VSD was compared among the following conditions: presence (+) and absence (-) of the collagen membrane, cover glass (thickness 120–170 μm), thinned skull (100–150 μm), and normal skull (450–500 μm). The small square in each box plot indicates the mean. Scale bars in (**a**) and (**b**), 1 mm.

For in vivo VSD imaging (Figure 1a), the implanted chamber was filled with a fluorescent dye (RH-795, Thermo Fisher Scientific, Waltham, MA, USA) dissolved at a concentration of 0.8 mg/mL in ACSF for 1.5 h. The dye solution was filtered before use (0.22 µm disposable sterile filter; Millipore, Eschborn, Germany). Following incubation with the dye, the cortex was thoroughly washed by repeatedly replacing the ACSF in the chamber. Then, the chamber was filled with fresh ACSF and covered with a glass cover slip. Brief electrical stimulation (0.6 mA, 1 ms) was delivered to the forepaw for each imaging trial, and forelimb-evoked neural activity was recorded as a fractional change in fluorescence (ΔF/F) using an optical recording system (Micam01, BrainVision, Tokyo, Japan) and a tandem-type epifluorescence microscope [21]. The fluorescence signals were recorded at 88 × 60 pixels and a 500 Hz frame rate. Fluorescent signals recorded from 16 consecutive trials with 16-s intervals were averaged to improve the signal to noise ratio. We used the Micam02 optical recording system (Brainvision) in the time-lapse imaging experiment (Figure 2) to assess dye diffusion characteristics across the collagen membrane, and the fluorescent images were captured every 1.5 min for 1.5 h. In an experiment to evaluate the optical transparency of the collagen membrane (Figure 2d), fluorescent signals were collected from 5000 pixels and averaged over five trials. The transmitted fluorescence was divided by the mean value obtained without the membrane, and the normalized fluorescence was then compared among in vivo imaging conditions. For pharmacological manipulation, 250 µM gabazine (G122500, Toronto Research Chemicals, North York, ON, Canada) dissolved in ACSF was use to fill the implanted chamber in order to block the γ-aminobutyric acid A (GABAA) receptor through the collagen membrane. Forelimb-evoked neuronal responses were compared before and 15 min after administering gabazine.

### 2.4. Histology

Following in vivo VSD imaging, the animals were anesthetized with pentobarbital (intraperitoneal) and transcardially perfused with 4% paraformaldehyde. The brain was removed, post-fixed in the same fixative for 24 h, and immersed in 30% sucrose solution. The brain was cut into 50 μm coronal sections and dye penetration into the cortex was confirmed using a digital microscope (BZ8100, Keyence).

## 3. Results

### 3.1. Chemical and Optical Properties of the Atelocollagen Membrane for VSD Imaging

To investigate use of the atelocollagen membrane for VSD imaging, we first confirmed dye (VSD RH-795) permeability and optical transparency of the membrane. VSD permeability was examined by monitoring time-lapse fluorescence changes in the plastic test tube (Figure 2a). The tube was separated into two parts by the collagen membrane; one side of the well was filled with dye and the other side was filled with ACSF. Sequential images of dye diffusion, and the time course of the fluorescence from the dye that penetrated the membrane are shown in Figure 2b,c, respectively. Dye fluorescence appeared in the ACSF side near the collagen membrane within 10 min of adding the dye; it diffused into the ACSF solution over time (Figure 2b). The fluorescence value (ΔFn) detected in the ACSF solution was saturated at approximately 30 min after the dye was added (Figure 2c), although the 30-min time courses were different between the two test samples. The collagen membrane sheet allows the passage of molecules smaller than few tens of thousands of Daltons (manufacturer’s unpublished data). Therefore, the dye may have diffused easily through the membrane, since popular VSDs are organic compounds with a low molecular weight (e.g., 585.42 for RH-795 dye). Next, optical transparency of the membrane was evaluated by measuring the fluorescent intensity of the dye; the dye was adsorbed with filter paper and a collagen membrane sheet was placed on it (Figure 2d). A comparison of fluorescence intensity values with and without the collagen membrane showed that transmitted light decreased to 70% due to the membrane when the RH-795 dye (excitation and emission maxima at 530 and 712 nm, respectively) was used. However, there was little difference in optical transparency between the collagen membrane, cover glass, and thinned skull, the latter of which are generally used for in vivo optical imaging (Figure 2d). These results suggest that the chemical and optical transparency characteristics of the atelocollagen membrane are competent for use in VSD imaging.

### 3.2. In Vivo VSD Imaging of the Sensory Response through the Implanted Window

**Figure 3:**
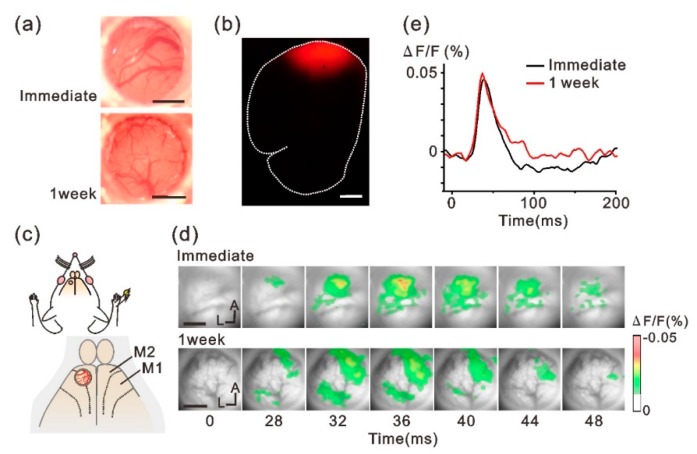
Forelimb-evoked VSD responses recorded through the implanted-cranial window. (**a**) Representative images of the brain surface in different rats obtained immediately (upper) and 1 week (lower) after the implant surgery. (**b**) Histological fluorescent assessment of VSD diffusion into the cortex through the collagen membrane. Red areas indicate the cortex stained by the VSD-RH795. Dotted line indicates the contour of the brain section. (**c**) Schematic view of the experiments. The implanted device was placed over M2. (**d**) Forelimb-evoked neuronal responses were recorded immediately (upper panels) and 1 week (lower panels) after implanting the chamber device. Data are from different animals. (**e**) Representative time course of the optical signal evoked by forelimb stimulation. Scale bars in (**a**,**b**,**d**), 1 mm. M1, primary motor cortex; M2, secondary motor cortex; A, anterior; L, lateral.

The tube-shaped cranial chamber device was implanted over the rat M2 cortex and in vivo VSD imaging was conducted to monitor neuronal activity (Figure 1). The photographs in Figure 3a show the cortical surface observed through the implanted chamber immediately (upper image) and 1 week (lower image) after the implantation surgery. Blood vessels running over the cortical surface were clearly seen even 1 week after implantation. The M2 region below the implanted chamber was stained by filling the chamber with the RH-795 dye solution. Figure 3b shows a fluorescence image from the brain section that was examined following completion of the experiments. Fluorescence was widely observed under the implanted chamber (red areas in Figure 3b), indicating that the RH-795 dye had smoothly penetrated the collagen membrane in the in vivo condition and successfully stained the cortex.

Following staining, forelimb-evoked neuronal responses were monitored in M2 through the collagen membrane (Figure 3c). We chose the M2 region for this study because this cortex represents a reproducible sensory response to forelimb stimulation [23]. Figure 3d,e show the spatiotemporal activity patterns and the time course of the forelimb-evoked responses in M2, respectively. The evoked optical responses were clearly detected through the collagen membrane in both conditions (Figure 3d, immediately: upper panels, 1 week: lower panels). In addition, the time course and amplitude of the optical signals were similar regardless of the time from implant surgery (Figure 3e), suggesting that the collagen membrane maintained the exposed cortex in good condition for 1 week.

**Figure 4:**
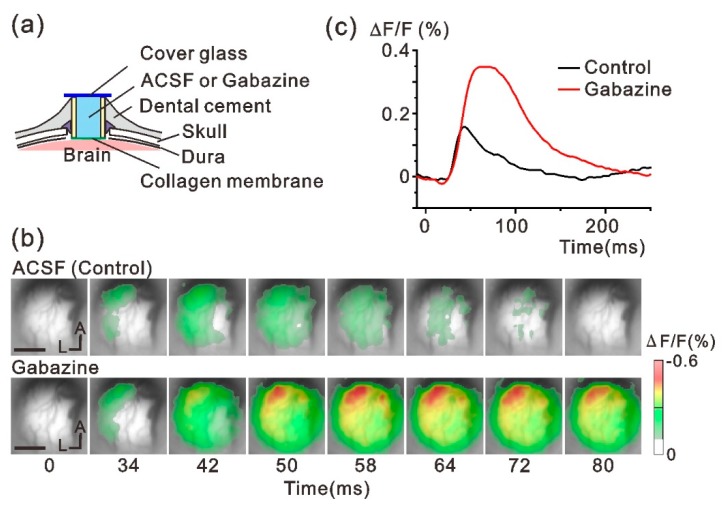
Pharmacological modulation of neuronal responses through the collagen membrane. (**a**) Schematic view of the experiment. The implanted tube device was filled with ACSF (control) or gabazine dissolved in ACSF. (**b**) Forelimb-evoked neuronal activity before (upper panels) and after administering gabazine (lower panels). (**c**) Representative time course of optical signals collected from the same brain regions before and after gabazine was administered. Scale bar in (**b**), 1 mm. A, anterior; L, lateral.

The implanted chamber was further examined for pharmacological manipulation of neuronal activity. We applied the GABAA receptor antagonist gabazine in the chamber (Figure 4a) and confirmed whether the drug modulated neuronal activity after passing through the collagen membrane. Figure 4b shows optically mapped forelimb-evoked neuronal responses before and after gabazine administration. The evoked M2 response was enhanced and spatially extended after gabazine was administered. The peak amplitude of the optical signal was more than twice the value of the control (Figure 4c), indicating that our implantable device was compatible with the pharmacological experiments. Specifically, manipulation of the neural network conditions or neural responsiveness was easily achieved by filling the chamber with any reagent, which might be an advantage as an implantable neural sensing device.

## 4. Discussion

The objective of the present study was to apply a collagen membrane as an artificial dura substitute for VSD imaging. The membrane had superior characteristics in chemical and optical transparency and maintained the cortical surface in good condition after implantation. Previous VSD imaging studies failed to stain brain tissue beyond the artificial dura [16,17]; this is the first study to report successful VSD imaging, where the brain was stained through an artificial dura that was previously implanted as part of a neural sensing device. There are currently no available VSDs for human application. However, once safety issues regarding VSDs are resolved [24], neural probing using VSDs should be considered as a next-generation BMI approach for human patients. Even in research using animals, the pharmacological manipulation ability of our device will be useful when conducting long-term VSD imaging studies.

The utilization of VSD signals may have potential as a new BMI framework. For example, VSD signals report inhibitory as well as excitatory neural activity, with positive and negative values, respectively [25,26]. To date, spike-based BMI methods cannot take advantage of this neural activity inhibition. Furthermore, VSD signals reflect subthreshold membrane dynamics in neuronal dendrites [27,28]. Probing dendritic processing may provide a different quality of information previously unattainable by neuronal firing analyses. Thus, voltage imaging data are expected to provide useful information that was unavailable in conventional BMI systems.

The cortical reaction to implantation of the artificial dura has been described in previous studies [16,29]. Over a span of months, previous studies observed cortical neovascularization and growth of thin white tissue, considered to be proliferation of the leptomeninges, between the dura substrate and the brain surface. After 1 week of implantation, no such symptoms were observed in our experiments, and the cortical surface seemed sufficiently healthy to report optical signals. However, as we did not obtain data after 1 week, future studies will evaluate the possible cortical reaction that could prevent long-term VSD imaging over the course of months.

The present study evaluated the collagen dura membrane in experiments using anesthetized rats. Therefore, our next step is to perform VSD imaging in freely behaving animals. As the implantable cranial window device was fabricated using a 3D printer, it will be easy to modify the outer shape to fit the fiberoptic periscope/microscope terminus described previously [8,9,30]. VSD signal quality is often degraded by movement-related artifacts [30]; however, our collagen membrane adhered closely to the cortical surface, which may help to reduce movement artifacts. Applying the collagen membrane to non-human primates is another necessary challenge toward future BMI configurations incorporating optical techniques.

Another direction of future research is a combination with optogenetics. After replacing the native dura with a transparent dura, several studies have already reported successful injection of a virus and transdural illumination through the artificial dura [29,31]. Because our membrane is thinner (35 μm vs. 100–200 μm), the viral injection procedure with glass pipettes should be easier. As any access to the brain, including penetration with electrodes and pipettes, damages cortical tissue, we are also planning to investigate any possible side effects of the collagen membrane that might promote tissue regeneration based on its hemostatic, chemotactic, and angiogenic properties [32]. Femtosecond-based multiphoton imaging is another important potential application of the implantable device. However, the detailed optical properties of the collagen membrane, such as dispersion and refractive index, must be evaluated prior to implementation.

## 5. Conclusions

In conclusion, the present study successfully demonstrated a new type of implantable neural sensor that enables visualization of cortical neural activity with the help of VSD probes. The sensor device employs an atelocollagen membrane as a material element for contacting the brain. Due to the chemical and optical transparency of the membrane, this device may potentially be utilized to develop future optical BMI applications.
